# Y-junction carbon nanocoils: synthesis by chemical vapor deposition and formation mechanism

**DOI:** 10.1038/srep11281

**Published:** 2015-06-11

**Authors:** Er-Xiong Ding, Jing Wang, Hong-Zhang Geng, Wen-Yi Wang, Yan Wang, Ze-Chen Zhang, Zhi-Jia Luo, Hai-Jie Yang, Cheng-Xiong Zou, Jianli Kang, Lujun Pan

**Affiliations:** 1 State Key Laboratory of Separation Membranes and Membrane Processes, School of Material Science and Engineering, Tianjin Polytechnic University, Tianjin 300387, China; 2 School of Physics and Optoelectronic Technology, Dalian University of Technology, Dalian 116024, China

## Abstract

Y-junction carbon nanocoils (Y-CNCs) were synthesized by thermal chemical vapor deposition using Ni catalyst prepared by spray-coating method. According to the emerging morphologies of Y-CNCs, several growth models were advanced to elucidate their formation mechanisms. Regarding the Y-CNCs without metal catalyst in the Y-junctions, fusing of contiguous CNCs and a tip-growth mechanism are considered to be responsible for their formation. However, as for the Y-CNCs with catalyst presence in the Y-junctions, the formation can be ascribed to nanoscale soldering/welding and bottom-growth mechanism. It is found that increasing spray-coating time for catalyst preparation generates agglomerated larger nanoparticles strongly adhering to the substrate, resulting in bottom-growth of CNCs and appearance of the metal catalyst in the Y-junctions. In the contrary case, CNCs catalyzed by isolated smaller nanoparticles develop Y-junctions with an absence of metal catalyst by virtue of weaker adhesion of catalyst with the substrate and tip-growth of CNCs.

Carbon nanocoils (CNCs) have received considerable attention owing to their unique helical morphology and their potential as functional materials, such as electromagnetic wave absorbers^1^, field-emitters^2^, mechanical springs^3^, electrodes in supercapacitors^4^, and resonators^5^. CNCs are generally prepared by chemical vapor deposition (CVD), which involves transition metal elements (*e.g.*, Fe, Co, and Ni) usually doped with other elements as a catalyst, and the most commonly used carbon source is acetylene. A certain amount of additives, such as indium and tin, is frequently added to promote the coil formation^6^,^7^. The nonwetting characteristic of In and Sn on the CNC surface induces repulsive interaction between the elements and the nanostructure, facilitating the coil growth^7^,^8^. In addition, H_2_S can modify the catalyst nanoparticles and make the catalyst composition variable, leading to asymmetric geometry and chemical constitution of the particles, as well as coil formation^9^.

Morphologies of carbon nanomaterials are of great significance because their morphologies play key roles in determining their physical and chemical properties, as well as potential applications. Fascinating branched morphologies of carbon nanomaterials, including carbon nanotubes and carbon nanofibers, together with their formation mechanisms have been reported^10^,^11^,^12^, and the Y-junction^13^,^14^ is one of them. Multibranched carbon nanotubes can be applied to create branched heterojunctions or complex multiple chemical sensors in one unit^11^, while the Y-junction ones are suitable for the fabrication of logic devices^14^, molecular-scale electronic devices and biological systems by virtue of their remarkable transduction capability^15^. In the field of CNC growth, many research targets have already been realized under certain experimental conditions, such as perpendicular arrays^16^, high yield^17^, controllable growth^18^, theoretical simulations^19^, and high-purity deposits^20^. For instance, Wu *et al.*^19^ studied the stretching instability and reversibility of tightly wound helical carbon nanotubes by atomistic simulations, and Wang *et al.*^20^ achieved nearly 100% pure CNCs with copper nanoparticles produced by atomic layer deposition. Moreover, various mechanisms have been proposed to illustrate the formation of CNCs, and it is generally accepted that the distinct carbon extrusion rates on different catalyst facets, namely catalytic anisotropy, give rise to the helical morphology of CNCs^21^,^22^. In addition to the abovementioned nonwetting characteristic of In/Sn and H_2_S-modified catalysts bringing about coil formation, regular insertion of pentagon-heptagon pairs at the junctions can also cause the formation of coiled single-walled carbon nanotubes^23^. More recently, Jian *et al.*^24^ raised the origin of catalytic anisotropy, that is growing tendency and rates of carbon deposition on the facets, edges and vertices of the catalyst grain. Morphologies of CNCs vary from group to group on account of the diverse fabrication approaches. Reports on the mirror symmetric V-shaped CNCs (V-CNCs) whose formation are ascribed to symmetric growth on the mirror planes of one catalyst nanoparticle are frequently found^25^,^26^,^27^. Nevertheless, the Y-junction CNCs (Y-CNCs) were merely provided by a few groups^27^,^28^. Coincidentally, these researchers held the same view that the formation of Y-CNCs was attributed to three branches stemming from one junction without offering more explanations. However, this interpretation is unspecific, and further insight into the morphology and formation mechanism is urgently needed. As with Y-junction carbon nanotubes, Y-CNCs can also be applied to switch and logic applications due to the peculiar Y-junction. Nonetheless, to the best of our knowledge, no literature specific to the synthesis and formation mechanism of Y-CNCs has been reported to date.

In this paper, we focus on the synthesis of Y-CNCs through CVD using Ni catalyst prepared by spray-coating method. A field-emission scanning electron microscope (FE-SEM) and a transmission electron microscope (TEM) were employed to observe the morphologies and structures of Y-CNCs. Element analysis of Y-CNCs was conducted using a energy dispersive X-ray spectrometer (EDX). The CNC fusing, nanoscale soldering/welding of CNCs, tip-growth and bottom-growth mechanisms are put forward to illustrate the formation mechanisms of Y-CNCs. We believe that this study would offer a valuable reference for the understanding of the synthesis and formation mechanism of Y-CNCs, thereby arousing great interest in the novel morphologies of carbon nanomaterials.

## Results and Discussion

**Figure 1:**
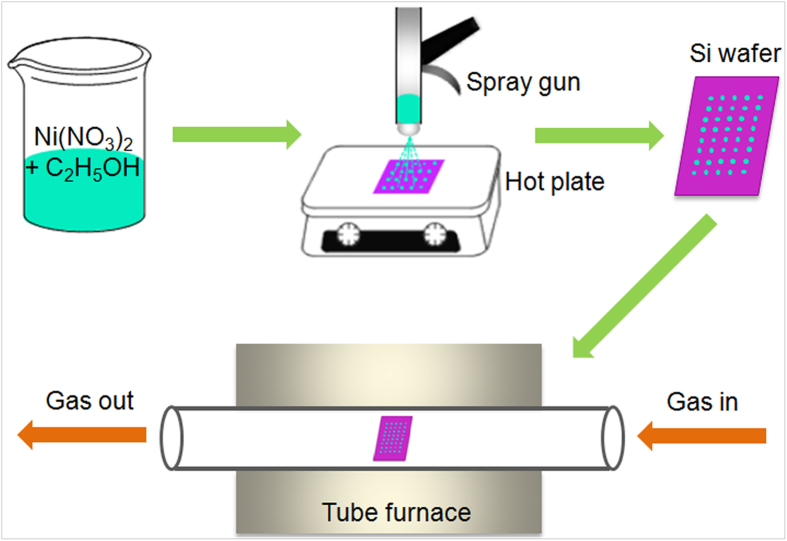
Schematic description of operational processes for Y-CNC synthesis.

**Figure 2:**
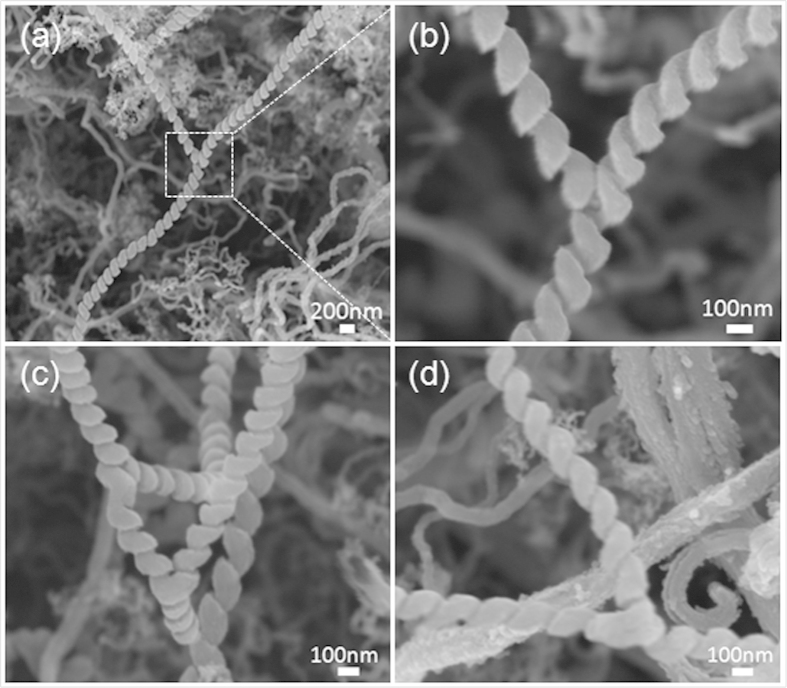
SEM images of Y-CNCs grown from the catalyst prepared by spray-coating two times.

The operational processes for Y-CNC synthesis are described in Fig. 1. Y-CNCs with average line diameter and coil pitch approximating 150 and 50 nm, respectively, are clearly shown in Fig. 2. In this case, the catalyst was prepared by spray-coating of the catalyst precursor solution two times. The morphology and distribution of catalyst nanoparticles were described in our previous paper^29^. The junction parts which seem to be welds in Fig. 2c,d are much more likely to be the backside images of the junction in Fig. 2b (an enlarged image of the part indicated by a square in Fig. 2a). It is very likely that this kind of junction is formed by inserting of the end of one CNC trunk to another. Further mechanism analysis of Y-CNC formation is given below.

**Figure 3:**
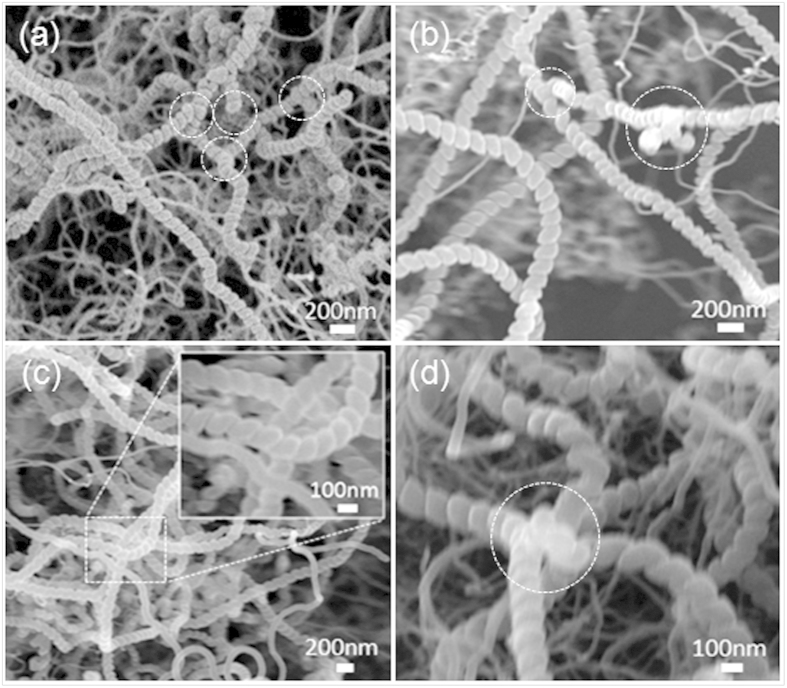
SEM images of Y-CNCs grown from the catalyst prepared by spray-coating four times. The parts labeled by white circles in the images are Y-junctions.

Y-CNCs with average line diameter and coil pitch of approximately 100 and 10 nm, respectively, are displayed in Fig. 3, and here the spray-coating time for preparation of the catalyst precursor film is four times. The parts labeled by white circles in the SEM images are all Y-junctions. Obviously, as seen from the SEM images, it is found that the number of CNCs as well as the Y-junctions in Fig. 3 is much larger than that in Fig. 2, indicating higher yield of the CNCs and Y-junctions in the sample whose spray-coating time for catalyst preparation is four times.

Morphological changes of the catalyst nanoparticles are considered to account for the difference in yield. Increased spray-coating time results in more catalyst nanoparticles with large size and regular shape, catalyzing the formation of more CNCs as shown in Fig. 3^24^,^29^. It is speculated that there might exist metal element contained in these junctions. Detailed explanation of Y-CNC formation is discussed later.

**Figure 4:**
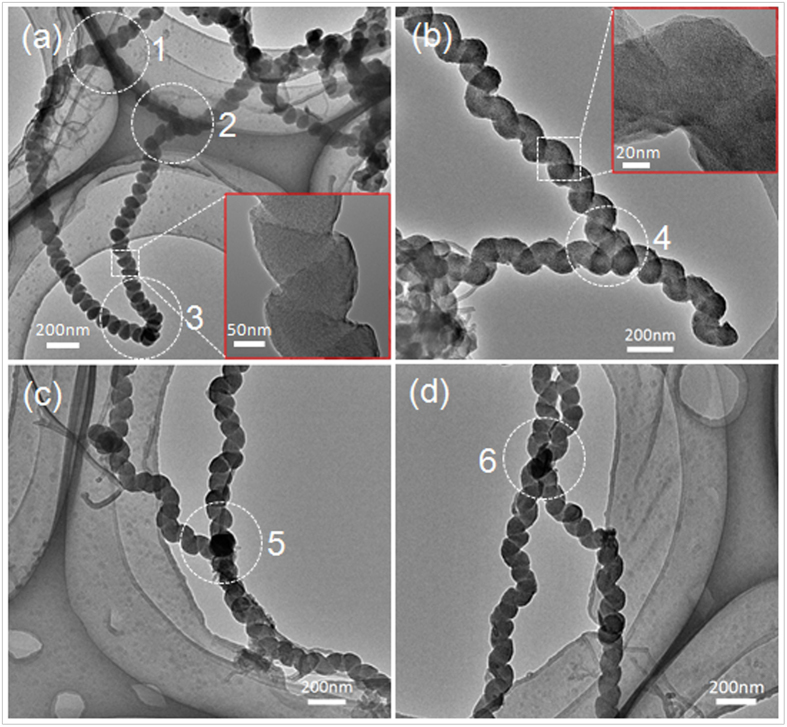
TEM images of Y-CNCs (a–c) and X-CNCs (d). The parts labeled by white circles in the images are Y-junctions. Spray-coating time for catalyst preparation of (**a**) and (**b–d**) are two and four times, respectively.

**Figure 5:**
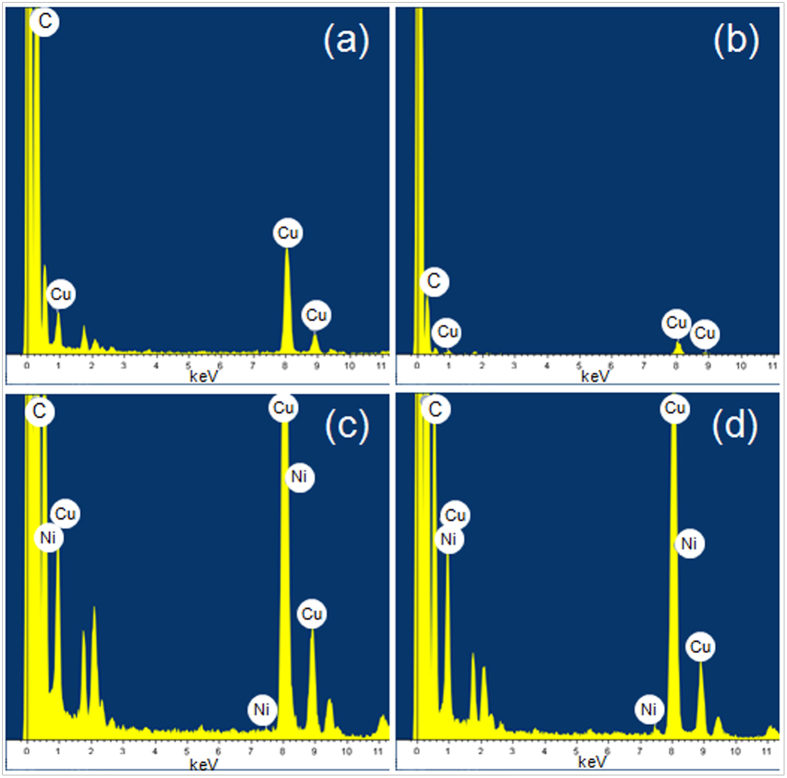
EDX characterization of CNCs. (**a**) and (**b**) are from the Y-junctions labeled by two circles (1 and 2) in Fig. 4a and circle 4 in Fig. 4b, respectively. (**c**) and (**d**) are corresponding to circle 5 in Fig. 4c and circle 6 in Fig. 4d, respectively.

TEM images of Y-CNCs are exhibited in Fig. 4. The CNCs in Fig. 4a and Fig. 4b–d are correspondingly from the same samples as those in Figs. 2 and 3, respectively, with similar average line diameter and coil pitch. The Y-CNCs indicated by white circle 1 and 2 in Fig. 4a are free of metal catalyst, though with blurring view in that the Y-CNCs are located on the edge of micropores of the TEM grid. Furthermore, EDX detection of the Y-junctions labeled by two circles (1 and 2) in Fig. 4a demonstrates that there is no metal element in the junctions (Fig. 5a and Table 1). The formation of the V-CNCs indicated by circle 3 in Fig. 4a is attributed to folding of the CNC trunk, and the darker area is the overlap of coils. Owing to the same color contrast of the whole Y-CNCs in Fig. 4b, it is surmised that no metal catalyst exists in the Y-junction. Moreover, EDX analysis of circle 4 verifies that there is no metal element in the Y-junction (Fig. 5b and Table 1). The Y-junction in Fig. 4b matches well with those displayed in Fig. 3a and the enlarged image in Fig. 3c. Seen from the high-resolution TEM images inserted in Fig. 4a,b, it is learned that the CNCs both from two and four times are coiled wires with a solid core rather than hollow ones. Black parts in the circles (5 and 6) can be seen at the junctions of Y-CNCs and X-shaped CNCs (X-CNCs) shown in Fig. 4c,d, respectively, and EDX detection confirms Ni presence in these junctions (Fig. 5c,d). Only 0.10 wt% and 0.21 wt% of Ni content can be detected (Table 1), which may be attributed to covering of Ni peak by Cu peak resulting from appearances of Ni and Cu at adjacent location.

**Figure 6:**
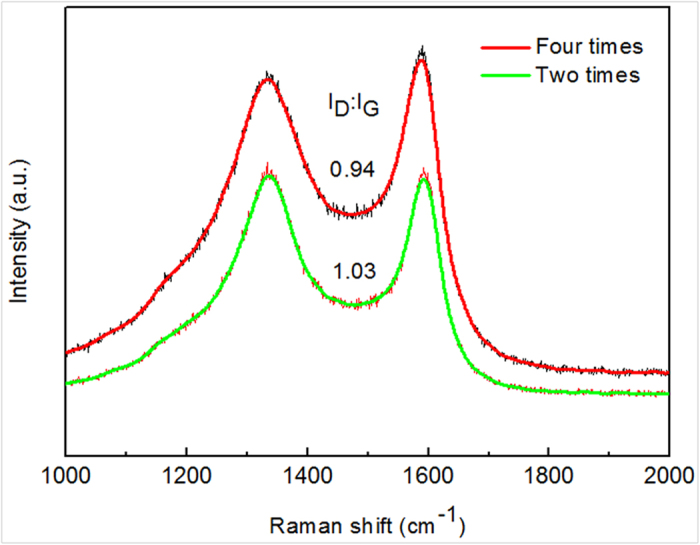
Raman spectra of CNCs.

The samples were inspected by Raman spectra with an excitation laser wavelength of 532 nm. Fig. 6 shows Raman spectra of CNCs from catalyst prepared by spray-coating time of two and four times. There exhibits two main peaks in the Raman spectra, one is around 1330 cm^−1^ known as D-band originated from structural defects (amorphous carbon, sp^3^ hybridization carbon and other impurities) in carbon materials, and the other is around 1590 cm^−1^ known as G-band originated from graphite structure (sp^2^ hybridization carbon). Obviously, the large peaks of D-band and the broad peaks of G-band in spectra indicate low graphitization of carbon nanomaterials in both samples. However, compared with the ratio of I_D_/I_G_ (1.03) from carbon nanomaterials using catalyst prepared from spray-coating time of two times, the ration of I_D_/I_G_ is lower from that of four times, implying there are more graphite structures in the latter case. Additionally, owing to more CNCs in the latter case (Fig. 3), it is concluded that these CNCs possess relatively more graphite structures. The obtained results from Raman spectra here is corresponding to those from previous reports in which Tang *et al.*^30^ demonstrated that CNCs from larger catalyst nanoparticles had higher level of graphitization.

**Figure 7:**
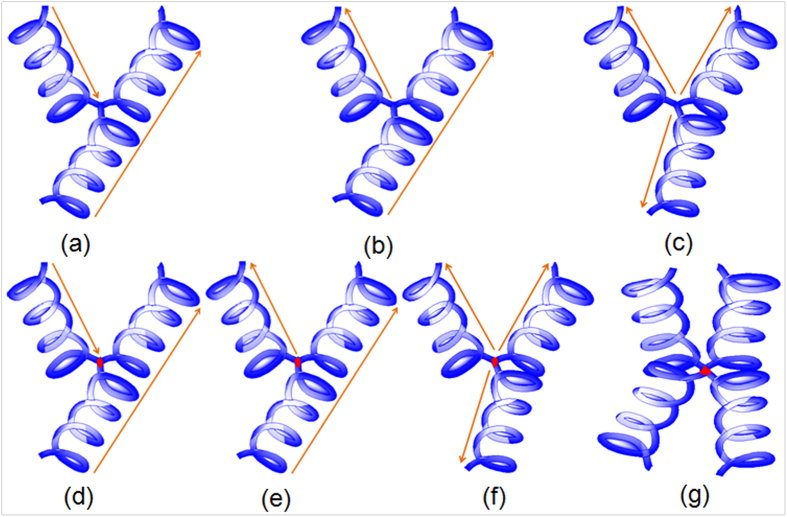
Schematic description of proposed growth models of Y-CNCs. Arrows and points represent growth direction of CNC branches and metal catalysts, respectively.

To explicate the formation mechanisms of the presented Y-CNCs, several growth models are demonstrated in Fig. 7. Arrows and points in the junctions represent growth directions of CNC branches and metal catalysts, respectively. Generally, two growth mechanisms, namely tip-growth and bottom-growth, are taken into account. Fig. 7b,c, and e,f are classified into tip-growth and bottom-growth, respectively. There is no metal catalyst in the junctions of Y-CNCs in Fig. 7a,b. With regard to Fig. 7a, the formation of this Y-junction can be ascribed to fusing of the end of one CNC stem to the trunk of another. It is clearly seen from Fig. 2b that the Y-CNCs are most likely formed by inserting the end of one CNC stem into the middle part between two units of a coil, followed by fusing *via* interaction of carbon atoms, and the fusing area covered by subsequent carbon deposits may be composed of sp^2^ and sp^3^ carbon atoms. The junction parts which seem to be welds in Fig. 2c,d further confirm the CNC fusing. This fusing phenomenon of carbon nanomaterials can also be found in branched carbon nano-structures^31^. Additionally, it was suggested that Y-shaped carbon nanotubes were formed by fusing of adjacent tubes as a result of the incremental growth of multiple graphene layers around the carbon nanotubes^32^. On the other hand, catalyst attachment on the sidewall of the CNC trunk may take place^33^; thereby, one CNC branch can derive from the attached catalyst, forming Y-CNCs. Given that the branched CNCs follow a tip-growth mechanism, then a metal catalyst will be supported to the tip, leading to absence of catalyst in the junction (Fig. 7b). In consequence,

the Y-CNCs in Figs 2 and 4a can be explained by the growth model shown in Fig. 7a,b, or both. As to the model in Fig. 7c, catalyst nanoparticles which are weakly adhered to each other can be divided during carbon extrusion; in the case of tip-growth, three CNC branches will extend along their own ways, causing formation of Y-CNCs without catalyst existing in the junction. According to our TEM observation, we conclude that it is less likely that tip-growth as is illustrated in Fig. 7b,c dominates in the case of two times. Instead, carbon fusing after occurrence of nanocoil collision may be dominant. The formation of Y-CNCs with catalyst absent in the junctions in Fig. 3a, the enlarged image in Figs 3c and 4b can be elucidated by the growth models in Fig. 7a–c.

It is the welding of the apex with a metal catalyst located on one CNC stem with the trunk of another that occurs in Fig. 7d, and the metal catalyst plays a key role in this case. Cui *et al.*^34^ also proposed the nanoscale soldering/welding mechanism to explain the formation of Y-junction single-walled carbon nanotubes using a molecular dynamics method, while Jia *et al.*^35^ adopted that mechanism to explain the formation of branched multi-walled carbon nanotubes. The aforementioned catalyst attachment on the sidewall of CNC trunk is reasonable for the model in Fig. 7e; however, it is bottom-growth of CNCs in this case. In relation to the model in Fig. 7f, it can be described as embranchment of three CNC branches from one agglomerated polyhedron catalyst with three catalytic facets, which is similar to previous reports^27^,^28^. Catalyst soldering/welding commonly emerges during formation of other branched carbon nanomaterials^11^,^30^,^35^,^36^. Similarly, bottom-growth of CNCs takes place in this case in which the polyhedron catalyst remains anchored to the substrate. The formation of the Y-CNCs with metal catalyst shown in Figs 3b–d and 4c can be explicated by the growth models exhibited in Fig. 7d–f. Nevertheless, four CNC branches may also originate from the agglomerated polyhedron catalyst provided that four catalytic facets are present, inducing X-CNCs in Fig. 7g, which can account for the formation of X-CNCs shown in Fig. 4d. In addition, there is another possibility, which is a superposition of several CNC trunks on one agglomerated catalyst nanoparticle that results in formation of X-CNCs. What type of growth model is correspondingly responsible for the formation of Y-CNCs displayed in our images, however, is unclear at the moment.

At the step of catalyst preparation, increasing spray-coating time makes larger and more regular nanoparticles. In regard to catalyst/substrate interaction, Si can chemically react with the metal catalyst at an elevated temperature, generating metallic silicide and a strong interaction^28^. Nonetheless, when a barrier layer such as silicon oxide in our cases is interlaid, the interaction will be weakened^37^. Typically, the difference of growth mode is explained in terms of adhesion force between the catalyst and the substrate. While a strong interaction favours the bottom-growth, a weak contact promotes the tip-growth^37^,^38^,^39^,^40^. Theoretically, the adhesion force should be proportional to the contact area, providing that other conditions are identical. For instance, Song *et al.*^38^ demonstrated that the interconnected catalyst islands which had strong contact with the substrate promoted bottom-growth, while tip-growth occured for the isolated catalyst nanoparticles that weakly interacted with the substrate. In our cases, consequently, the large interconnected catalyst islands are inclined to have stronger adhesion with the substrate, resulting in CNC following a bottom-growth mechanism and catalyst presence in the Y-junctions. On the contrary, the isolated smaller nanoparticles weakly anchored to the substrate can be lifted up during CNC formation, giving rise to tip-growth of CNCs and catalyst absence in the Y-junctions. Apart from the abovementioned explanations, further insight should be investigated. *In-situ* observation *via* TEM of the formation of Y-CNCs might be the optimal approach, regardless of the complicated operations and intractable experimental conditions.

In summary, Y-junction CNCs were synthesized by thermal CVD using Ni catalyst prepared by spray-coating method. Moreover, the possible growth mechanisms of Y-CNCs were explored according to the emerging morphologies of Y-CNCs. Overall, it can be divided into two situations: with or without metal catalyst in the Y-junctions of CNCs. Carbon fusing can account for the catalyst absence in the Y-junctions, while catalyst soldering/welding brings about catalyst presence in the Y-junctions. Apart from those, bottom-growth and tip-growth are also taken into account. Increasing spray-coating time leads to interconnected catalyst islands which have strong adhesion with the substrate, bringing about bottom-growth of CNCs and the presence of metal catalyst in the Y-junctions, whereas CNCs catalyzed by isolated smaller nanoparticles weakly adhering to the substrate follow a tip-growth mechanism, producing Y-CNCs which are free of metal catalyst in the junctions. Except for our proposed formation mechanisms, more specific growth mechanisms should be further investigated to understand the formation of Y-CNCs better.

## Methods

### Sample preparations

The experimental details are based on our previous study^29^. Typically, a catalyst precursor solution (0.01 mol/L) consisting of absolute ethanol and nickel nitrate (Ni(NO_3_)_2_·6H_2_O) was sonicated for 30 min. Next, the ethanol solution was spray-coated using an air brush pistol (Gunpiece GP-1) onto a 1 cm × 1 cm heated Si substrate (500 μm, p-doped, (100), 300 nm SiO^2^). Thereafter, the Si substrate with catalyst precursor film was inserted into a quartz tube (inner diameter, 70 mm) heated at 400 °C. Nickel nitrate was decomposed in air for 10 min followed by evacuation. 500 sccm Ar was filled in to atmospheric pressure till 650 °C, then mixed gases of H_2_/Ar (100/100 sccm) were flushed in. After reaching the target temperature of 750 °C in 10 min, 15 sccm C_2_H_2_ was introduced in for deposition, and 15 min later the furnace was switched off and allowed to cool to room temperature under 50 sccm Ar. Specific operational processes are shown in Fig. 1.

### Instrumental characterization

The surface morphologies of as-prepared deposits were examined by a field-emission scanning electron microscope (FE-SEM, Hitachi S-4800) with an accelerating voltage of 10 kV. A transmission electron microscope (TEM, JEM-2100) operated at 200 kV was employed to observe the structures of Y-CNCs. Additionally, element composition of Y-CNCs were analyzed using a energy dispersive X-ray spectrometer (EDX), while Raman spectra were gained via a Raman spectrometer (Renishaw) with a laser excitation wavelength of 532 nm.

## Additional Information

**How to cite this article**: Ding, E.-X. *et al.* Y-junction carbon nanocoils: synthesis by chemical vapor deposition and formation mechanism. *Sci. Rep.* **5**, 11281; doi: 10.1038/srep11281 (2015).

## Figures and Tables

**Table 1:** Relative contents of elements analyzed from EDX detection shown in Fig. 5.

		Figure 5a	Figure 5b	Figure 5c	Figure 5d
C	wt%	86.71	94.18	79.35	81.38
Cu	wt%	13.29	5.82	20.56	18.40
Ni	wt%			0.10	0.21
